# Modelling of Chest Wall Motion for Cardiorespiratory Activity for Radar-Based NCVS Systems

**DOI:** 10.3390/s20185094

**Published:** 2020-09-07

**Authors:** Anuradha Singh, Saeed Ur Rehman, Sira Yongchareon, Peter Han Joo Chong

**Affiliations:** 1 Department of Electrical and Electronic Engineering, Auckland University of Technology, Auckland 1010, New Zealand; saeed.rehman@flinders.edu.au (S.U.R.); peter.chong@aut.ac.nz (P.H.J.C.); 2 College of Science and Engineering, Flinders University, Adelaide 5042, Australia; 3 Department of Information Technology and Software Engineering, Auckland University of Technology, Auckland 1010, New Zealand; sira.yongchareon@aut.ac.nz

**Keywords:** chest wall motion, respiration, cardiac, simulation, mathematical modelling

## Abstract

Chest wall motion can provide information on critical vital signs, including respiration and heartbeat. Mathematical modelling of chest wall motion can reduce an extensive requirement of human testing in the development of many biomedical applications. In this paper, we propose a mathematical model that simulates a chest wall motion due to cardiorespiratory activity. Chest wall motion due to respiration is simulated based on the optimal chemical–mechanical respiratory control-based mechanics. The theory of relaxation oscillation system is applied to model the motion due to cardiac activity. The proposed mathematical chest wall model can be utilized in designing and optimizing different design parameters for radar-based non-contact vital sign (NCVS) systems.

## 1. Introduction

Continuous monitoring of vital signs, namely respiration and heartbeat, can provide information in predicting the undesired events like cardiac arrest, critical dizziness, arrhythmias, cardiac rhythm, temperature regulation, synchronization with respiration rate [1]. The continuous monitoring can be achieved either through contact (wearable) sensors or non-contact (radar) sensors. It is not practical to use wearable sensors 24/7 throughout the year. One of the alternative solutions for long term continuous monitoring is using radar-based sensors [2]. The monitoring of surface chest wall motion can provide vital signs information which can be useful in many applications e.g., designing a radar-based non-contact vital sign system for long term home health care and sleep monitoring. Another application of modelling chest wall motion due to respiratory signals is to study the radiotherapy for treating a lung-tumor. The main issue in radiotherapy is that a lung tumor may move due to respiration. From existing studies, it is observed that chest wall motion is associated with tumor movements; therefore, it can help in predicting the tumor position [3].

The radar-based system for vital sign detection is developed based on movements of a target, i.e., chest wall motion due to respiration and cardiac activity. Chest wall displacement with respect to time is reflected in the phase variation in the received signals. Subsequently, the modelling accuracy of non-contact vital sign (NCVS) systems depends on the modelling of chest wall motion with respect to time, i.e., the shape of the chest wall motion. The performance and testing of a robust design depend on the analysis and strength of the experimental dataset available. Though a larger and exhaustive dataset produces an opportunity for the design to be tested and analyzed in all possible scenarios, it would require a considerable number of real-time experiments. It not only consumes time, costs, and power but is also impractical for participating subjects. This can efficiently be managed through simulation and mathematical modelling. In earlier works related to radar-based systems, many researchers conducted exhaustive experiments for analyzing radar-received signals [2]. However, any change in design parameter or experimental scenarios like change in subject position, movement, and presence of multiple subjects, then reconducting all experiments are required. Additionally, fewer efforts have been made for modelling the chest wall motion due to respiration and heart activity.

In literature, most of the simulation for radar-based NCVS systems to date are based on a simplistic approach of assuming chest wall motion as sinusoidal [4,5]. The respiration model presented by Fouladi et al. [6] follows the sinusoidal pattern for a periodic breathing activity. The airflow was taken as sinusoidal, and consequently, the time integration of airflow produces the lung volume variation pattern during breathing activity which represents the chest wall movement [7]. However, the main issue is the cusp at the peak after inhalation, which offered a substantial variation of simulation wave with real signals. This inferred that the pure sinusoidal functional model suffers from significant deviation during real-life breathing and modelled breathing pattern. Another model based on the duty cycle, depending on the inhalation and exhalation phases was presented by Li et al. [8]. Primarily three models referenced to duty cycle are designed to model respiratory signals based on cosine function (CM) [9,10], the absolute value of cosine function (ACM) [11], and even power of cosine function (EPCM) [12,13,14]. The selection of the order of EPCM modelling determines the duty cycle, and there is a significant deviation of a duty cycle from the real signal for different values of the order of EPCM modelling [8]. To address these challenges, the power of the absolute value of the cosine function (PACM) [8] was used to model the breathing signal. Based on the dilation and erosion of sinusoidal PACM base wavelet, the shape of the waveform was adjusted empirically to produce breathing signal with better accuracy. However, the model was realized with a complex algorithm and with very high computational complexity. The mentioned challenges opened the domain of modelling the chest wall motion due to respiration to be simplistic, and computational inexpensive.

The heartbeat signal is complicated and nearly periodic. Almost all simulations to date are based on assuming the chest wall motion due to cardiac activity as sinusoidal [15,16,17,18], while Morgan et al.’s [19] model is based on heart functioning. A short impulsive action is initiated during the systolic phase when the heart ventricles start emptying. This impulsive motion is filtered by the bone and tissue structure to finally observe the chest wall motion [20,21]. Morgan et al. [19] chose a second-order Butterworth filter to emulate the bones and tissues structure. Further, the model was slightly simplified to avoid signal processing complexity by Weishaupt et al. [22]. However, as the heart rate variability is observed for cardiac activity, the chest wall displacement varies considerably with respect to time. This implies that the shape of chest wall motion pulse varies considerably and cannot be simplified to a simplistic sinusoidal model [23,24,25] and hence the design performance of the system cannot be analyzed comprehensively through a sinusoidal mathematical model. Addressing the mentioned issue, a mathematical model of chest wall movements due to cardiorespiratory activity is proposed in this paper.

Our proposed model is the first step toward modelling chest wall motion due to cardiorespiratory activity. This would help in customizing it for the various respiratory pattern that arises due to different health conditions/diseases. For example, Kaneko et al., in their study, assess the effect of posture, age, and gender on the breathing movements in healthy subjects [25]. The outcome of the study is helpful in evaluating breathing movement, which is a critical element in a physical therapy assessment. By mapping the simulation parameters for similar observations to study the effect of age, gender, and various disease scenarios, the presented study can be further extended. The chest wall motion can be used as a critical parameter in assessing patient’s health condition during treatment or post-surgery. For example, assessment of the change in chest expansion post-thoracotomy may help in the development of the patient-tailored chest physiotherapy [26]. In another study [27], Diaz et al. emphasized an evaluation of the changes in chest wall motion that occur in the patient with the neuromuscular disease during mechanical ventilation. It is demonstrated that measurement of chest wall motion can be useful in the determination of optimal ventilator setting for the children and young adults. A recent survey [28] highlights that change in vital sign can be an indicator of the infant’s physiology. The continuous analysis of vital sign can help in predicting disease like sepsis, bronchopulmonary dysplasia, brain injury, and mortality by increased vigilance and proactive involvements of the clinical practitioners. Thus, the simulation of chest wall motion can be extended further to be used in designing analytical tools and potential applications as discussed. However, this research work does not explicitly investigate those health conditions, and we will leave it for our future work.

The rest of the paper is organized as follows. The proposed mathematical model for the chest wall motion is described in Section 2. Section 3 presents the experimental analysis and discussion. With summary and future directions, the paper is concluded in Section 4.

## 2. Proposed Model

### 2.1. Respiration Signal

**Figure 1:**
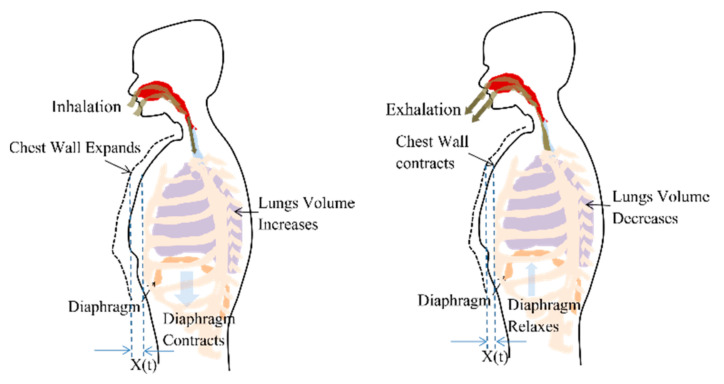
Chest wall motion resultant due to the contraction and relaxation of diaphragm causing the pleural pressure variation.

Respiration or breathing is a natural periodic activity which involves airflow into and out of lungs, also called pulmonary ventilation. The contraction and relaxation of the diaphragm cause pleural pressure variations and governs the airflow, as shown in Figure 1. The decrease in lung pressure causes an increase in lung volume. Considering the ribcage, chest wall and lungs, forming a rectangular prism shape, the increase in lung volume is accompanied by proportional chest wall movement [29]. Therefore, a chest wall movement x(t) follows the pattern of lung volume variations during a breathing cycle.

In this work, the respiration cycle is simulated based on the optimal chemical–mechanical respiratory control model, which has been utilized earlier to develop respiratory control simulator [30]. The neuro-mechanical effector that relates a neural respiratory output to a resultant mechanical airflow can be approximated by the electrical RC model based on the lumped parameter mode [31]. The equation of motion can be expressed as

(1) $$P\left(t\right)=\dot{V}\left(t\right).R_{rs}+V\left(t\right).E_{rs}$$

where *P*(*t*): Isometric respiratory pressure measured at FRC, $\dot{V}\left(t\right)$: instantaneous airflow, *V*(*t*): instantaneous lung volume, *R_rs_*: total respiratory system resistance, and $E_{rs}$ represents the elastance of the lung, chest wall, and airways. The model considers only two phases of breathing pattern as repeating inhale and exhale phases. The empirical equations for lung volume variation pattern and, consequently, the chest wall movements for a breathing cycle during inhalation and exhalation periods, can be derived as follows

(2) $$V\left(t\right)=\frac{\tau_{rs}}{R_{rs}}\left[A_{1}t^{2}+A_{2}t+A_{3}\left(1-e^{\frac{-t}{\tau_{rs}}}\right)\right]+V_{0}e^{\frac{-t}{\tau_{rs}}}\;0\leq t\leq t_{1}$$

(3) $$V\left(t\right)=\frac{P\left(t_{1}\right)}{R_{rs}.\left(\frac{1}{\tau_{rs}}-\frac{1}{\tau }\right)}\left[e^{\frac{\left(t-t_{1}\right)}{\tau }}-e^{\frac{-\left(t-t_{1}\right)}{\tau_{rs}}}\right]+V\left(t_{1}\right).e^{\frac{-\left(t-t_{1}\right)}{\tau_{rs}}}\;t_{1}\leq t\leq t_{1}+t_{2}$$

where *P*(*t*_1_) is the isometric pressure at time *t*_1_. $a_{0}$, $a_{1}$, and $a_{2}$ are pressure pulse shaping parameters and,

$$A_{1}=a_{2}$$

$$A_{2}=a_{2}-2a_{2}\tau_{rs}$$

$$A_{2}=a_{0}-a_{1}\tau_{\mathrm{rs}}+2a_{2}\tau_{\mathrm{rs}}{}^{2}$$

$$\tau_{\mathrm{rs}}=R_{\mathrm{rs}}.C_{\mathrm{rs}}$$

τ, the rate of decline of an inspiratory activity, $t_{1}$ and $t_{2}$ are inhale and exhale duration, respectively.

### 2.2. Cardiac Signal

The heart is placed in the space called a thoracic cavity. The thoracic cavity is protected by the thoracic wall or commonly called chest wall which consists of a rib cage and associated skin, muscle, and fascia. Inside the thoracic cavity, the heart is located atop the diaphragm having its apex closer to the anterior surface of the thoracic cavity. For every beat, the heart contracts and expands with the apex taping against the chest wall [32].

**Figure 2:**
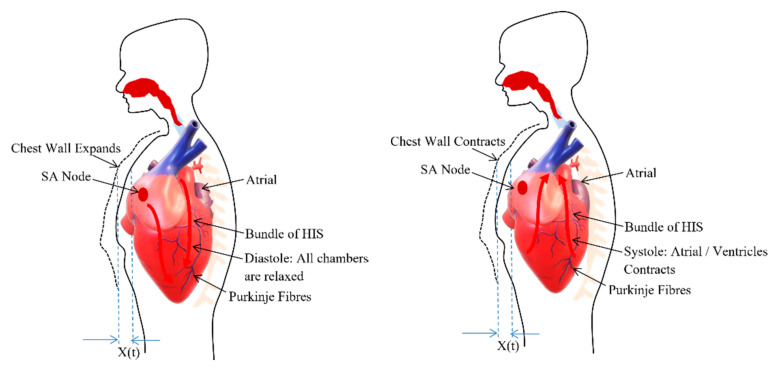
Chest wall motion resultant due to the relaxation (diastole) and contraction (systole) of chambers.

The heart consists of muscle tissue, commonly called fibers containing contractile elements and coordinate cardiac contraction. The coordination of mechanical activity of the chambers is requisite for the efficient working of the heart. During the systole and diastole, the contraction and expansion of the muscle tissue or contractile elements govern the thoracic wall or chest wall movement [33] and is described in the Figure 2. The systole and diastole are a rhythmic activity that describes the heart rate. The heart consists of its own rhythm generator called the sinoatrial (SA) node which comprises of a small strip of modified muscle tissue, about 20–34 mm, on the posterior wall of the atrium [32].

The cardiac impulse generated at the SA node is conducted by the contractile cells or the muscle tissues (myocardial cell) of the heart. Excitation–contraction coupling ties electrical depolarization to the mechanical expansion and shortening of myocardial cells [32]. The different branches derive from the bundle of His and conduct the impulse to the ventricles. This special conduction system consisting of muscle tissues coordinate the contraction of the heart. Through this conducting system, excitation is transferred to the heart muscle to coordinate its contraction from the apex upwards. This ensures the same spontaneous rate of depolarization to the other parts of the heart to have the same spontaneous rhythm [33].

The sinoatrial (SA) node (cardiac pacemaker) rhythmic behavior is represented by a relaxed oscillatory system [34]. It is an oscillatory system having a characteristic of adapting its intrinsic frequency to the frequency of external driving signal without changing its amplitude [35]. The oscillatory behavior of the SA node is defined as

(4) $$\frac{d^{2}x}{{\mathrm{dt}}^{2}}-\alpha \left(1-x^{2}\right)\frac{\mathrm{dx}}{\mathrm{dt}}+\omega^{2}x=0$$

The rhythm of the SA node cardiac pulse governs the contraction and expansion of contractile cells or muscle tissues. This muscle tissues directly controls the rhythm of thoracic or chest wall activity. The excitation and depolarization cycle of SA node is synchronized with the systole and diastole cycle of cardiac activity. The thoracic wall or chest wall observes expansion and contraction cycle in coordination with the systole and diastole cycle where its rhythmic behavior is synchronously represented by the SA node behavior. Therefore, the heartbeat model used in this study is represented by a relaxed oscillatory system.

For a NCVS system the heart rate is extracted from the phase variations in the reflected signal caused by chest wall motion. These chest wall motion has been modelled as linear sinusoidal motion in the earlier literatures assuming the heart rhythm as simple oscillatory behavior. However, the phase variation of radar reflected signal depends on the minute movement of chest wall and small variation in movement can result in large variation in the phase or resultant heart rate. Since, heart is treated as a network of elements and these elements show the oscillatory behavior that can be modelled as nonlinear van der pol oscillator, therefore, the chest wall motion has been modelled in rhythm to the rhythm of heart network element as novel approach for chest wall movement modelling.

## 3. Experimental Results and Discussion

We simulate chest wall motion pulse using Simulink, MATLAB 2019b. A sampling rate of 1 kHz is chosen to generate the simulation signals. The simulated waveforms are technically vetted through a separate experimental dataset available for the chest wall motion [36]. The dataset contains different trials obtained from 11 participants recorded in a supine position from a respiratory belt BIOPAC SS5LB attached to the subject’s chest in different scenarios such as free-breathing, breath-hold, post-exercise, and irregular breathing. The breath hold is used for cardiac activity and rest dataset for respiration. The dataset contains different trials for each participant (post exercise-5, free breath-16, irregular-11). Each participant’s data are compared with total number of generated simulated waves covering entire parameter range. The correlation coefficient of all the simulated wave with each participant is shown in the box plots. The median value for each participant indicates that the better similarity is observed with proposed simulated wave as compared to the sinusoidal model. For the comparison of our simulated waveforms and the waveforms from the dataset, the simulated waveforms are resampled to 100 Hz, and dataset waveforms are smoothened to arrive at a common comparison platform.

### 3.1. Simulation Result

#### 3.1.1. Chest Wall Motion—Respiration

**Figure 3:**
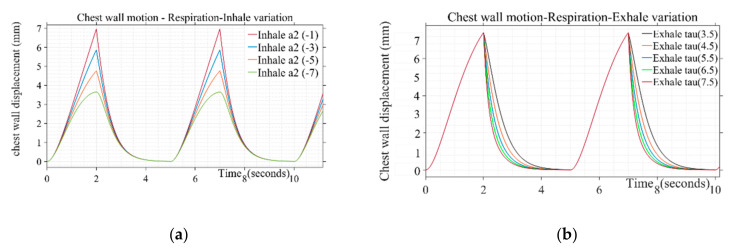
(**a**) Variation of inhale parameter on the chest wall motion due to respiration for τ: 4.5 (**b**) Variation of exhale parameter on the chest wall motion due to respiration for $a_{2}:-5$.

Design parameters including pulse duration ($t_{1}+t_{2})$ inhale shaping ($a_{2}$), exhale shaping (τ), and peak amplitude are varied to study their effect on simulated respiration wave shapes. Different values used in presented simulations are given in Table 1. The breathing pulse duration ($t_{1}+t_{2})$ can take a range from 4 to 5 s, which corresponds to the respiration rate of 12 to 16 breaths/min. Similarly, the peak amplitude parameter can range from 0.035 to 0.11, corresponding to the chest wall displacement from 4 to 12 mm [37]. The values for presented simulation are taken as steps from 0.035 to 0.07 corresponding to 4 to 7 mm of chest wall motion, respectively. The shape defining parameter for inhale ($a_{2})$ and exhale (τ) follows the range variation of −1 to −7 and 3.5 to 7.5, respectively. The inhale shape approaches a straight line resulting in a triangular shape of respiration pulse for inhale parameter ($a_{2}$) lower than −1 and starts following a circular pattern with a large duration cusp at the top beyond the parameter value of −7, which is not a realistic scenario [30]. The step increment is taken to generate different simulated shapes covering the entire range for the pulse shaping parameter. Similarly, air discharge during exhalation follows smooth discharging as a type of exponential decay [31]. Beyond the mentioned parameter range from 3.5 to 7.5, the shape adopts a triangular straight-line decay and discontinuity at the transition stage from “end of exhale” to “rest” and then “start of inhale” phase for next cycle. Figure 3a,b depicts a variation of inhalation- and exhalation-shaping parameters on the chest wall waveshape due to breathing. The shape of the wave during the inspiratory phase is found to be more concave upward indicating the loaded breathing condition through variation of the parameter $a_{2}$. The parametric variation of τ simulates the rate of decline of inspiratory activity, i.e., for the sharper rate of decline with a longer pause between exhale and next inhale instance for the same duty cycle wave represents the breathing pattern of the post-exercise phenomenon.

#### 3.1.2. Chest Wall Motion—Cardiac

Design parameter for simulation of chest wall motion due to cardiac activity is given in Table 2. The parametric variation of **α** simulates the behavior of atrial sinus (SA) node and atrial ventricular (AV) node during biological rhythms for different human activities such as sleeping, wake cycle etc. Similarly, the pulse duration and maximum chest wall displacement of the wave can be simulated through variation of $\omega^{2}$ and peak amplitude parameters, respectively.

**Figure 4:**
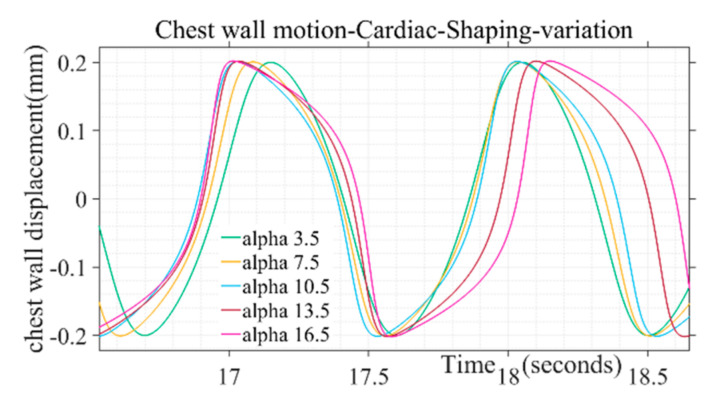
Variation of alpha pulse shape parameter on chest motion due to cardiac activity.

The shape of the simulated chest wall displacement during heart activity was found to be more bulged out in the middle through higher values of the parameter (α) as illustrated in Figure 4. For chest wall simulation, cardiac pulse duration parameter is taken in the range from 50 to 110 in incremental steps. The parameter range corresponds to the heart rate 60 to 100 beats/min for a healthy adult. Similarly, the peak amplitude parameter ranges from 0.2 to 0.5, corresponding to the chest wall displacement range from 0.2 to 0.5 mm [38]. The shape defining parameter (α) for chest wall motion follows the range variation of 3.5 to 16.5, respectively. Beyond the mentioned parameter range the wave shape adopts a trapezoidal form and discontinuity while approaching half of the chest motion amplitude during a beat cycle which is not in tandem with actual chest wall motion where no discontinuity is observed for a beat cycle [37]. Single-step increments are taken to generate different simulated shapes covering the entire range for the pulse shaping parameter.

### 3.2. Validation and Comparision Analysis

During a normal breath, chest wall motion comprises of two components, the displacement from respiration activity and second, the displacement due to cardiac activity. However, if a chest wall displacement is captured during a breath-hold condition, the displacement is caused by heart activity only. The motion due to heart activity is too small (0.2–0.5 mm) as compared to the motion due to respiration (3–12 mm). Therefore, a dataset of chest wall motion due to heart activity only is extracted from the breath-hold duration and used to perform the similarity analysis.

**Figure 5:**
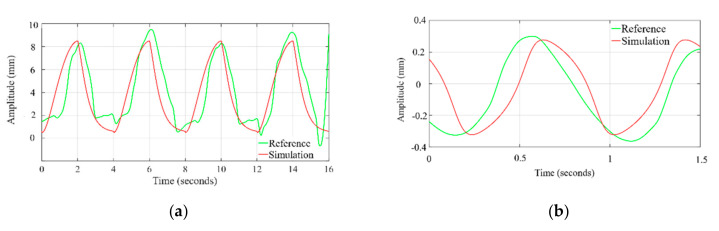
Time profile comparison of chest wall motion due to (**a**) respiration and (**b**) heartbeat, with dataset.

The time profile of the chest wall movement due to cardiorespiratory activity obtained from the simulation is compared with the human chest wall experimental data [36]. The data at channel 2, where the respiratory belt (SS5LB) is connected to measure the chest motion, are plotted, and zoomed in time axis to visually compare with the simulation waveform. As shown in Figure 5a the exhale–inhale pattern closely follows the experimented dataset wave. The pulse duration and in turn, the respiration rate is the same for both the simulated and referenced signal. As demonstrated in Figure 5b, the simulated systole–diastole cycle closely follows the experimented dataset wave. Our proposed model produces accurate matching between the duty cycle of the actual cardiac signal to the model generated wave. The similarity between simulated waves and the signals obtained from the referenced cardiac-respiratory dataset is demonstrated through the Pearson product-moment correlation coefficient and dynamic time warping (DTW). The Pearson product-moment correlation coefficient for two signals A(t) and B(t) is identified as a similarity index and is defined as:

(5) $$\rho \left(A,B\right)=\frac{1}{N-1}\left(A_{i}-\mu_{A}\sigma_{A}\right)\left(B_{i}-\mu_{B}\sigma_{B}\right)$$

where $\mu_{A}$ and $\sigma_{A}$ are the mean and standard deviation of *A*, respectively, and $\mu_{B}$ and $\sigma_{B}$ are the mean and standard deviation of *B*, with each variable has *N* scalar observations. In time series analysis, the normalized time-dependent Pearson correlation coefficient is an indicator of similarity index as the cross-correlation coefficient value must lie in the range from −1 to 1, with one indicating perfect correlation [39,40].

Additionally, in time series analysis, another technique dynamic time warping (DTW) is used for measuring the similarity between two temporal sequences [41]. DTW works on the principle of stretching the vectors to make them similar, and minimum possible distance between vectors is calculated for stretching. This distance is a parameter to measure the similarity of two sequences. We have used this distance to compare the similarity of simulated and sinusoidal chest wall motion with experimental and dataset chest wall motion.

#### 3.2.1. Comparison of Respiration Simulation Signal

**Figure 6:**
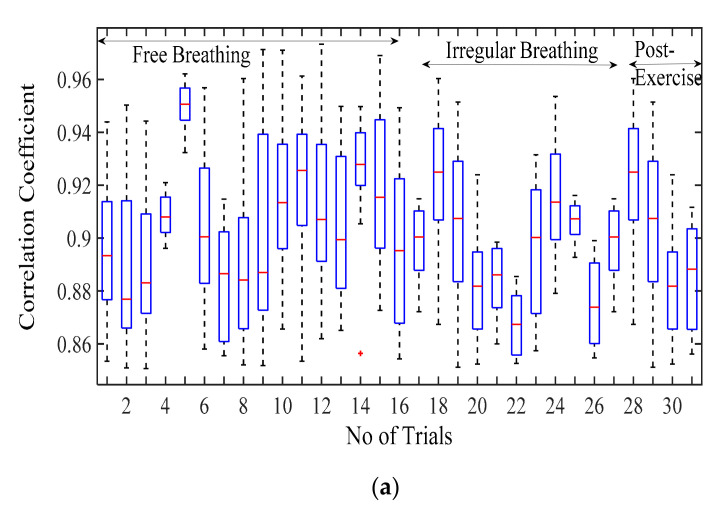

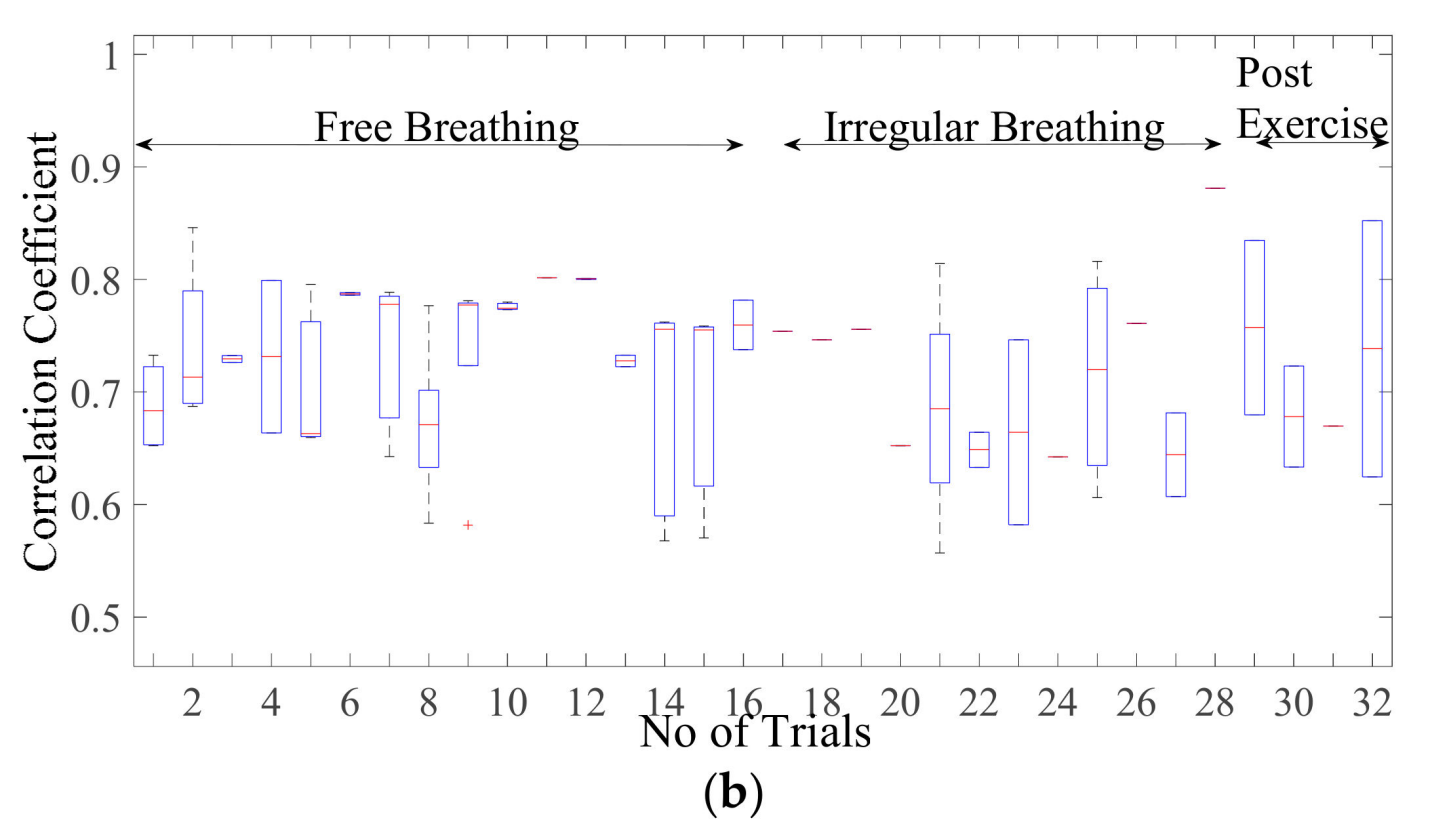
Correlation coefficient for the free breathing, irregular breathing, and post-exercise breathing with (**a**) simulated wave and (**b**) sinusoidal wave.

Figure 6a displays the correlation coefficient for different breathing scenario for the subjects lying in a supine position. For the free-breathing scenario, it is observed that the maximum correlation coefficient approaches 0.97 with most of the values for coefficient lying between a range of 0.9 and 0.97. The mean cross-correlation coefficient of the simulation-dataset wave is in a range of 0.86–0.94, the 75th percentile reaching above 0.9 on average. A similar pattern can be observed for irregular and post-exercise breathing as well. However, the similarity index between a sinusoid and dataset waveforms lies in the range of 0.68–0.84 with the median lying in the range of 0.65–0.77 correlation coefficient, as shown in Figure 6b. This indicates that the chest wall displacement due to respiration is convincingly represented through proposed simulation model in comparison to the assumption of sinusoidal motion.

**Figure 7:**
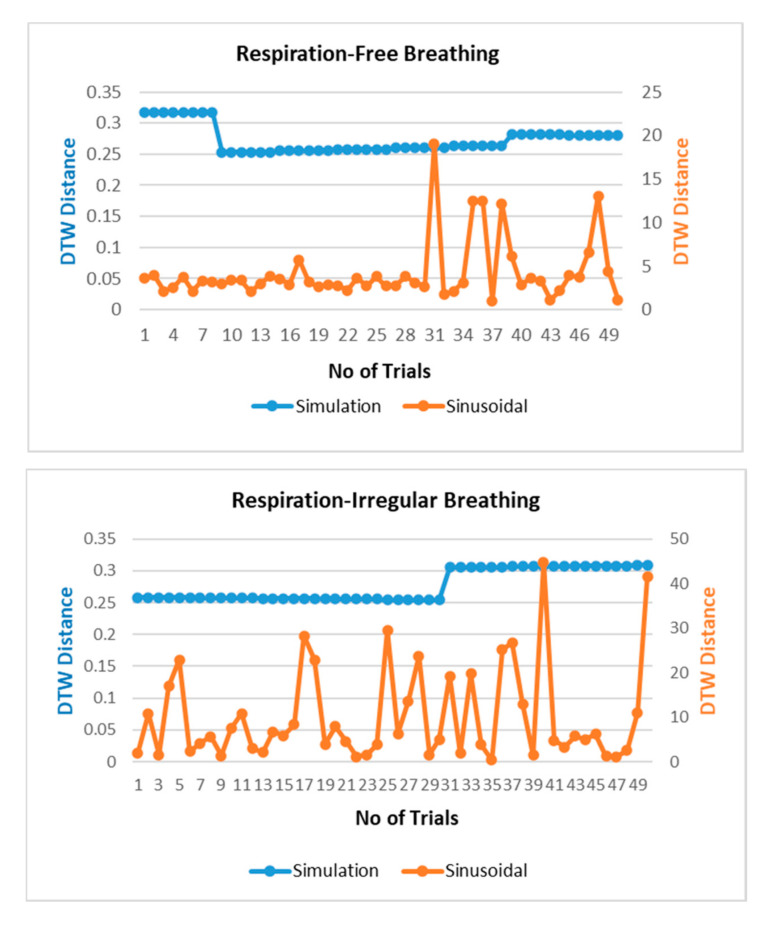

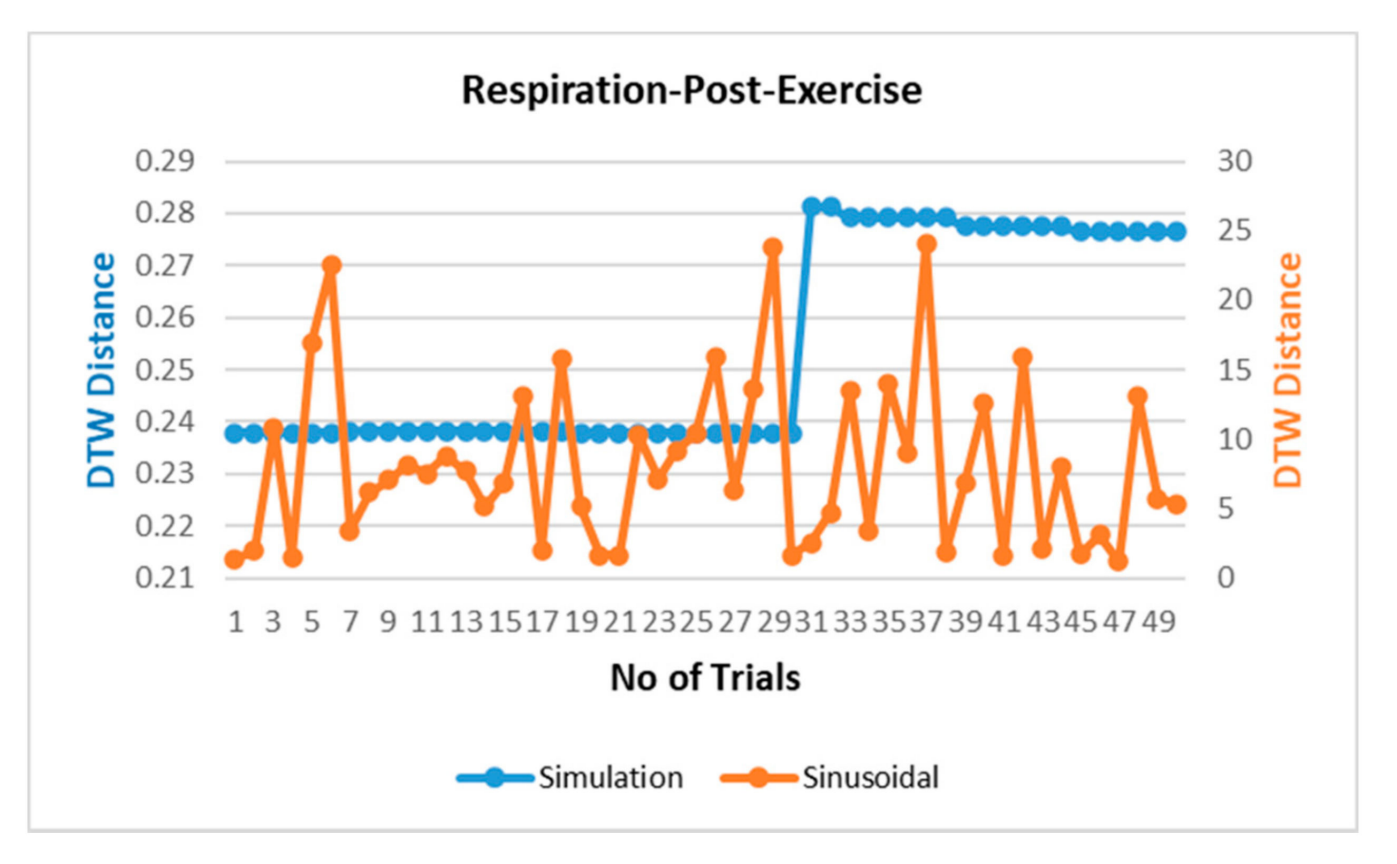
Comparison of dynamic time warping (DTW) between simulation-dataset and sinusoidal-dataset waves.

The respiration waves are nonlinear time series in nature, and hence, revalidation of the simulated model is performed through dynamic time warping, a nonlinear similarity matching technique for two time series. The distance between two waves acquired through DTW analysis is shown in Figure 7 for different breathing types. The DTW distances between proposed simulation and experimental datasets waves lie at nearly zero between the range of 0.2 and 0.3 while the DTW distance for sinusoidal simulation lies in the range of 2–15. From the results in Figure 7, the dynamic warping distance between the simulated and experimental wave is consistent and matches closely.

#### 3.2.2. Comparison of Cardiac Simulation Signal

**Figure 8:**
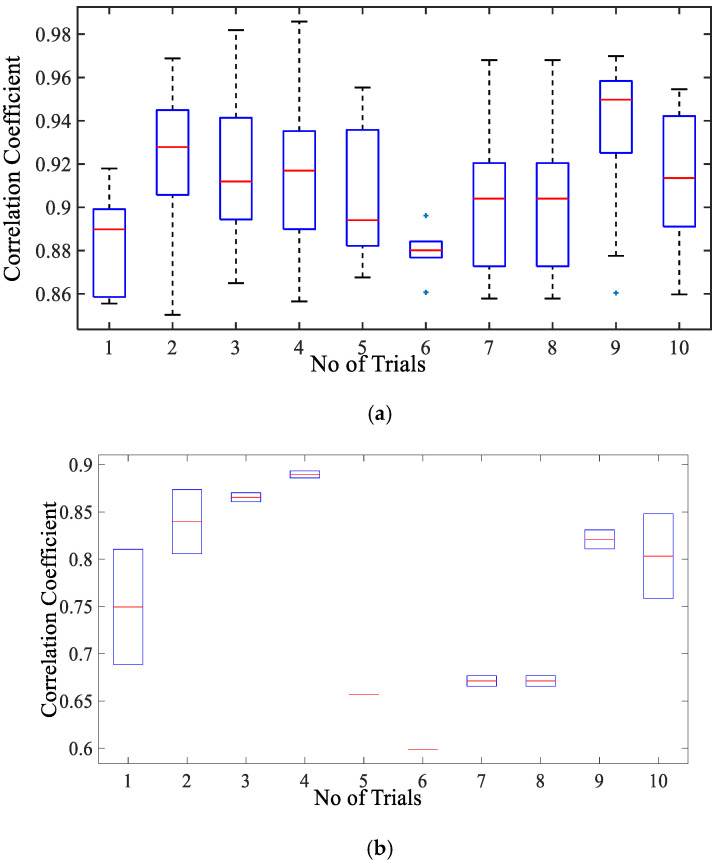
Box-plot representation of correlation coefficient for the chest wall motion due to cardiac activity with (**a**) simulated wave and (**b**) sinusoidal wave.

Figure 8a presents the correlation coefficient for cardiac activity (breadth hold condition). It is observed that the median of the correlation coefficient for simulation with reference to the cardiac dataset is around 0.93, the 75th percentile is above 0.9. The maximum correlation coefficient achieved is in a range of 0.9–0.98. The inter-quartile range is also implying that the similarity between the two signal waves is quite high. Whereas the similarity index for sinusoidal wave shape is much lower as compared to simulated wave shape lying in the range of 0.59–0.89 with a median in the range of 0.7–0.84 as given in Figure 8b. This infers that simulated shape represents the actual chest wall displacement due to heart activity.

**Figure 9:**
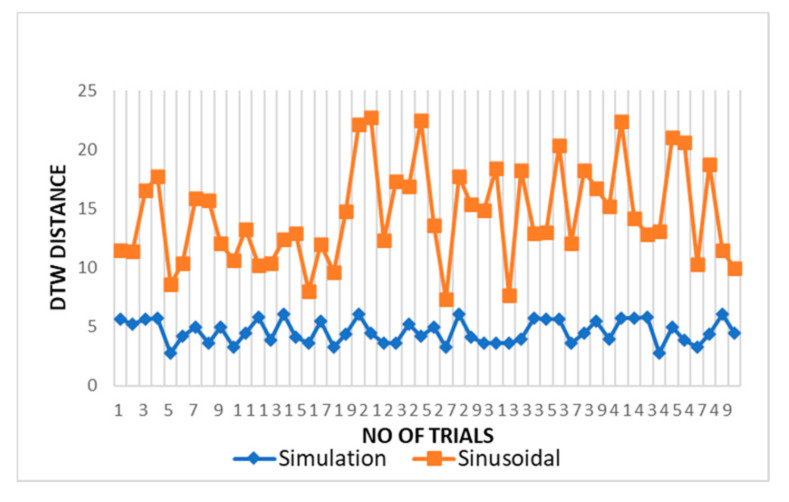
Comparison of DTW between cardiac simulation-dataset and sinusoidal-dataset waves.

The DTW distance between simulated and experimental dataset waves for cardiac activity acquired through DTW analysis is shown in Figure 9 for different trials. The threshold distance for DTW similarity match was taken through the experimental cardiac waves match data samples. The DTW distances between proposed simulation and experimental datasets waves closely approach one and lie between range of 2 and 5 with respect to the DTW distance for sinusoidal simulation, which lies in the range of 10–25. From the results in Figure 9, the dynamic warping distance between simulation and experimented wave is consistent and matches closely.

## 4. Conclusions

This work contributes to the development of a mathematical model for ubiquitous and nonobtrusive cardio-respiratory vital sign monitoring through radar-based NCVS system. The comparative analysis through cross-correlation demonstrates that the simulated chest wall motion is in close agreement with the real motion and hence validate the applicability of simulated chest motion in cardiac-respiratory applications. The cross-correlation coefficient between the simulated waveform and the experimental dataset approaches to the range of 0.95–0.97, indicating a high degree of similarity of both time sequences.

Our proposed model is not only useful for validating the design parameters through a target modelling for radar-based NCVS system applications but can also be utilized for the development of techniques to evaluate the cardio-respiratory parameters of chest surface motion beneficial in validating the design of artificial cardio-respiratory systems. The proposed model demonstrated the ability to simulate the varying chest wall motion in different scenarios such as free-breathing and post-exercise breathing etc.

The limitation of our study is that it relies on the single experimental dataset available for the chest wall motion. However, in future work, exhaustive experiments with a varying subject profile in terms of age, gender, weight, medical history can be employed for the measurement of chest displacements. This chest wall measurement may be compared with more simulation waveforms to arrive at a conclusion for a shape defining condition of diseases such as lung tumor, sleep apnea or different disease triggering scenarios. In addition, these simulation parameters may be employed for designing a care system to address the diseases or scenarios without exhausting experimentation of the design over real subjects.

## Figures and Tables

**Table 1:** Design parameter range for respiration wave used in simulation.

Pulse Duration ($t_{1}+t_{2})$	4, 5
Inhale shaping ($a_{2}$)	(−1), (−3), (−5), (−7)
Exhale shaping (τ)	3.5, 4.5, 5.5, 6.5, 7.5
Peak amplitude	0.035, 0.045, 0.05, 0.06, 0.07

**Table 2:** Design parameter range for cardiac wave used in simulation.

Peak amplitude	0.2, 0.5
Pulse Duration $\left(\omega^{2}\right)$	50, 60, 70, 80, 90, 110
Pulse Shaping (α)	3.5, 7.5, 10.5, 13.5, 16.5
